# μ-(Acetic acid)-di-μ-chlorido-bis[tri­phenyl­tellurium(IV)] monohydrate

**DOI:** 10.1107/S160053681301739X

**Published:** 2013-06-29

**Authors:** Feng Hu, Chao Xu, Hua-Tian Shi, Qun Chen, Qian-Feng Zhang

**Affiliations:** a Institute of Molecular Engineering and Applied Chemistry, Anhui University of Technology, Ma’anshan, Anhui 243002, People’s Republic of China; b Department of Applied Chemistry, School of Petrochemical Engineering, Changzhou University, Jiangsu 213164, People’s Republic of China

## Abstract

The asymmetric unit of the title compound, C_38_H_34_Cl_2_O_2_Te_2_·H_2_O, contains two independent Te^IV^ cations, each coordinated by three phenyl ligands, two Cl^−^ anions and one acetic acid mol­ecule in a distorted octa­hedral C_3_Cl_2_O geometry; the longer Te⋯Cl distances ranging from 3.2007 (11) to 3.4407 (11) Å and the longer Te⋯O distances of 3.067 (3) and 3.113 (3) Å indicate the weak bridge coordination. The Cl^−^ anion and acetic acid mol­ecule bridge the two independent Te^IV^ cations, forming the dimeric complex mol­ecule, in which the Te⋯Te separation is 3.7314 (4) Å. In the crystal, the water molecules of crystallization link the Te^IV^ complex mol­ecules into chains running along the *b-*axis direction *via* O—H⋯O and O—H⋯Cl hydrogen bonds.

## Related literature

For background to organotelluronium salts: see: Collins *et al.* (1988 ▶); Oilunkaniemi *et al.* (2001 ▶); Ziolo & Extine (1980 ▶); Ziolo & Troup (1979 ▶); Zhou *et al.* (1994 ▶). For related structures, see: Jeske *et al.* (1996 ▶); Oilunkaniemi *et al.* (2001 ▶). For a description of the Cambridge Structural Database, see: Allen (2002 ▶).
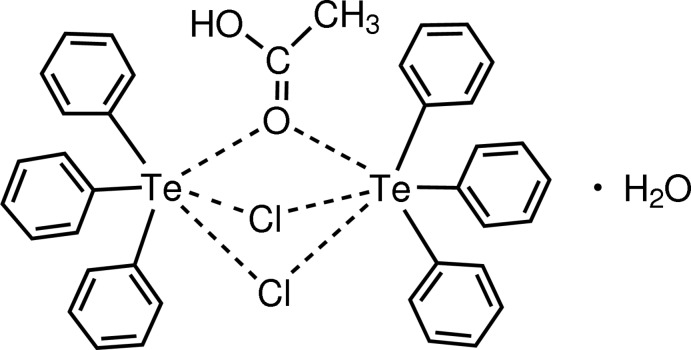


## Experimental

### 

#### Crystal data

C_38_H_34_Cl_2_O_2_Te_2_·H_2_O
*M* *_r_* = 866.77
Monoclinic, 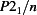

*a* = 13.9469 (6) Å
*b* = 9.3616 (4) Å
*c* = 27.7941 (12) Å
β = 96.584 (1)°
*V* = 3605.0 (3) Å^3^
*Z* = 4
Mo *K*α radiation
μ = 1.80 mm^−1^
*T* = 296 K
0.22 × 0.15 × 0.12 mm

#### Data collection

Bruker SMART APEXII CCD area-detector diffractometer
Absorption correction: multi-scan (*SADABS*; Bruker, 2001 ▶) *T*_min_ = 0.692, *T*_max_ = 0.813
23122 measured reflections
8145 independent reflections
6494 reflections with *I* > 2σ(*I*)
*R*_int_ = 0.033

#### Refinement

*R*[*F* ^2^ > 2σ(*F* ^2^)] = 0.032
*wR*(*F* ^2^) = 0.078
*S* = 1.08
8145 reflections
407 parameters
H-atom parameters constrained
Δρ_max_ = 0.85 e Å^−3^
Δρ_min_ = −0.51 e Å^−3^

### 

Data collection: *APEX2* (Bruker, 2007 ▶); cell refinement: *SAINT* (Bruker, 2007 ▶); data reduction: *SAINT*; program(s) used to solve structure: *SHELXTL* (Sheldrick, 2008 ▶); program(s) used to refine structure: *SHELXTL*; molecular graphics: *SHELXTL*; software used to prepare material for publication: *SHELXTL*.

## Supplementary Material

Crystal structure: contains datablock(s) I, global. DOI: 10.1107/S160053681301739X/xu5713sup1.cif

Structure factors: contains datablock(s) I. DOI: 10.1107/S160053681301739X/xu5713Isup2.hkl

Additional supplementary materials:  crystallographic information; 3D view; checkCIF report

## Figures and Tables

**Table 1:** Selected bond lengths (Å)

Te1—C11	2.129 (3)
Te1—C21	2.124 (3)
Te1—C31	2.116 (3)
Te1—Cl1	3.2366 (9)
Te1—Cl2	3.4407 (11)
Te1—O1	3.067 (3)
Te2—C41	2.129 (4)
Te2—C51	2.126 (4)
Te2—C61	2.118 (4)
Te2—Cl1	3.2802 (9)
Te2—Cl2	3.2007 (11)
Te2—O1	3.113 (3)

**Table 2:** Hydrogen-bond geometry (Å, °)

*D*—H⋯*A*	*D*—H	H⋯*A*	*D*⋯*A*	*D*—H⋯*A*
O2—H2*A*⋯O1*W*	0.84	2.13	2.972 (5)	174
O1*W*—H1*W*⋯Cl2^i^	0.88	2.38	3.205 (4)	155
O1*W*—H2*W*⋯Cl2^ii^	0.87	2.41	3.200 (4)	152

Symmetry codes: (i) 
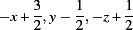
; (ii) 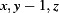.

**Fig. 1.**
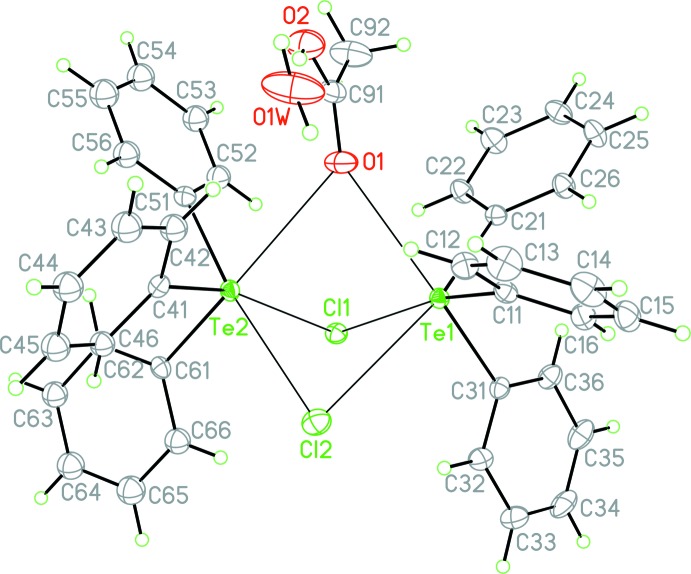
The structure of the title compound (µ-Cl)2(µ-CH3COOH)(Ph3Te)2.H2O, showing the atom-numbering scheme and displacement ellipsoids at the 50% probability level. The Te···O and Te···Cl secondary bonds were drawn in lines.

**Fig. 2.**
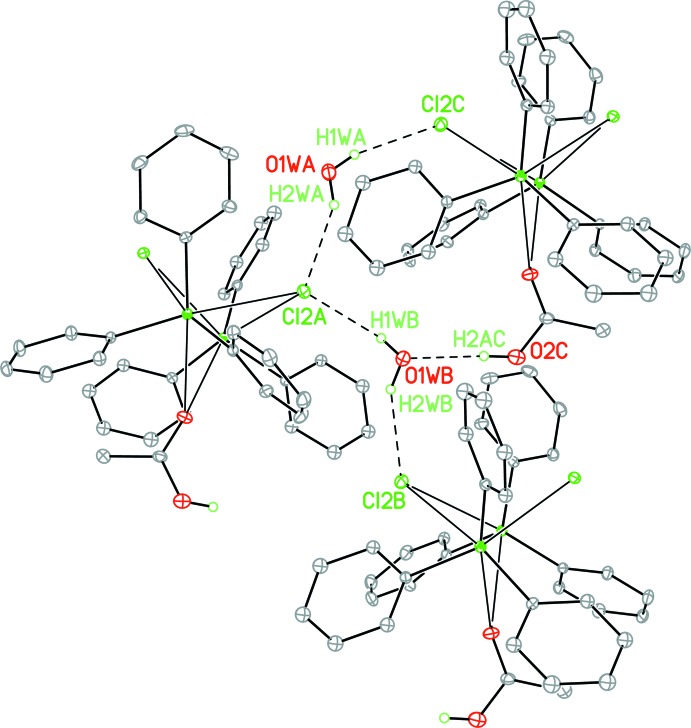
The intermolecular O—H···Cl and O—H···O hydrogen-bonds (dash lines) are displayed in the crystal lattice.

## supplementary crystallographic information

### Comment

Organotelluronium salts, R_3_TeX, have attracted considerable interest because of their application in organic synthetic chemistry (Zhou *et al.*, 1994). In the past several decades, a large number of triorganotelluronium salts have been prepared and many of their structures have been determined. Previous studies on such triorganotelluronium salts have shown that the salts have relatively complex structures due to weak bonding interactions between the tellurium atom and the anion (Ziolo & Extine, 1980). It has become evident that the interactions are sensitive to the nature of both them. Moreover, the structural features are also influenced by the organic groups and the presence or absence of solvent of crystallization (Ziolo & Troup, 1979). The X-ray structure determinations of several (Ph_3_Te)X (X = halide, SCN^-^, NCO^-^, [NO_3_]^-^, ^1^/_2_[SO_4_]^2-^, ^1^/_2_[Hg_2_Cl_6_]^2-^, ^1^/_2_[PtCl_6_]^2-^, ^1^/_2_[IrCl_6_]^2-^ and [AuCl_4_]^-^) salts have established that in the solid state the structural features are governed by weak secondary tellurium-anion interactions which may result in the trigonal pyramidal geometry around tellurium into a five- or six-coordinate entity (Collins *et al.*, 1988; Oilunkaniemi *et al.*, 2001; Ziolo & Extine, 1980; Ziolo & Troup, 1979). In this paper, we report the structural characterization of bis(µ_2_-chloride)-(µ_2_-acetic acid-*O*)- bis(triphenyltelluronium) hydrate monosolvate which is expected to expand the pool of the known organotelluronium chemistry.

The structure of the title compound, (µ-Cl)_2_(µ-CH_3_COOH)(Ph_3_Te)_2_.H_2_O (HAc = CH_3_COOH), consists of two Ph_3_Te^+^ cations, two chloride anions, one acetic acid molecule and one water molecule linked by a complex network of Te···Cl and Te···O secondery bonds and hydrogen bonds into infinate chains. The geometry around the tellurium atom is pseudo-octahedral, with three phenyl groups, two chloride atoms and one oxygen atom from the acetic acid. The two Ph_3_Te^+^ cations occupy on the opposite trigonal faces of octahedra, as shown in Fig. 1. The two tellurium atoms form two secondary bonds of 3.068 (4) and 3.113 (4) Å invoving the oxygen atom of the acetic acid molecule, which are longer than those in (Ph_3_Te)_2_SO_4_.5H_2_O (av. 2.797 (9) Å) (Collins *et al.*, 1988), but are still shorter than the sum of the van der Waals radii of the tellurium and oxygen atoms. The two bridging Te···Cl distances involving non-hydrogen-bonded Cl(1) atom are almost equal (3.236 (3) and 3.279 (3) Å), while those involving hydrogen-bonded Cl(2) atom are inequal (3.199 (3) and 3.439 (3) Å). The average Te···Cl distances of 3.288 (3) Å in the title compound is in the range of the van der Waals radii of the tellurium and chloride atoms. The Ph_3_Te^+^ cation in the title compound has its expected structure as well as normal distances and angles (Allen, 2002), for example, the six Te—C bond lengths in the two cations are normal and have a mean value 2.124 (4) Ph_3_Te^+^ (Jeske *et al.*, 1996). The [(µ-Cl)_2_(µ-HAc)(Ph_3_Te)_2_] moieties are further linked by two kinds of the intermolecular hydrogen bonds of (H_2_O)O—H···Cl (av. O···Cl = 3.205 (4) Å) and (HAc)O—H···O(H_2_O) (O···O = 2.962 (2) Å), forming one-dimensional infinate chains (see Fig. 2).

### Experimental

Ph_3_TeCl (212 mg, 0.55 mmol) in water (5 mL) was added into a hot aqueous solution (5 mL) containing the acetic acid (69%, 0.025 mL, 0.22 mmol). A pale brown precipitate was obtained almost immediately. The precipitate was filtered, washed with water and Et_2_O, and dried. Recrystallization from acetone-water (1:1) at room temperature afforded brown block crystals suitable for X-ray diffraction. Yield: 140 mg (57%).

### Crystal data

**Table:**

C_38_H_34_Cl_2_O_2_Te_2_·H_2_O	*F*(000) = 1704
*M**_r_* = 866.77	*D*_x_ = 1.597 Mg m^−^^3^
Monoclinic, *P*2_1_/*n*	Mo *K*α radiation, λ = 0.71073 Å
Hall symbol: -P 2yn	Cell parameters from 2274 reflections
*a* = 13.9469 (6) Å	θ = 2.0–23.6°
*b* = 9.3616 (4) Å	µ = 1.80 mm^−^^1^
*c* = 27.7941 (12) Å	*T* = 296 K
β = 96.584 (1)°	Block, brown
*V* = 3605.0 (3) Å^3^	0.22 × 0.15 × 0.12 mm
*Z* = 4	

### Data collection

**Table:**

Bruker SMART APEXII CCD area-detector diffractometer	8145 independent reflections
Radiation source: fine-focus sealed tube	6494 reflections with *I* > 2σ(*I*)
Graphite monochromator	*R*_int_ = 0.033
phi and ω scans	θ_max_ = 27.5°, θ_min_ = 1.5°
Absorption correction: multi-scan (*SADABS*; Bruker, 2001)	*h* = −15→18
*T*_min_ = 0.692, *T*_max_ = 0.813	*k* = −11→12
23122 measured reflections	*l* = −36→36

### Refinement

**Table:**

Refinement on *F*^2^	Primary atom site location: structure-invariant direct methods
Least-squares matrix: full	Secondary atom site location: difference Fourier map
*R*[*F*^2^ > 2σ(*F*^2^)] = 0.032	Hydrogen site location: inferred from neighbouring sites
*wR*(*F*^2^) = 0.078	H-atom parameters constrained
*S* = 1.08	*w* = 1/[σ^2^(*F*_o_^2^) + (0.0323*P*)^2^ + 1.0404*P*] where *P* = (*F*_o_^2^ + 2*F*_c_^2^)/3
8145 reflections	(Δ/σ)_max_ = 0.001
407 parameters	Δρ_max_ = 0.85 e Å^−^^3^
0 restraints	Δρ_min_ = −0.51 e Å^−^^3^

### Special details

**Table:**

Geometry. All esds (except the esd in the dihedral angle between two l.s. planes) are estimated using the full covariance matrix. The cell esds are taken into account individually in the estimation of esds in distances, angles and torsion angles; correlations between esds in cell parameters are only used when they are defined by crystal symmetry. An approximate (isotropic) treatment of cell esds is used for estimating esds involving l.s. planes.
Refinement. Refinement of F^2^ against ALL reflections. The weighted R-factor wR and goodness of fit S are based on F^2^, conventional R-factors R are based on F, with F set to zero for negative F^2^. The threshold expression of F^2^ > 2sigma(F^2^) is used only for calculating R-factors(gt) etc. and is not relevant to the choice of reflections for refinement. R-factors based on F^2^ are statistically about twice as large as those based on F, and R- factors based on ALL data will be even larger.

### Fractional atomic coordinates and isotropic or equivalent isotropic displacement parameters (Å^2^)

**Table:**

	*x*	*y*	*z*	*U*_iso_*/*U*_eq_	
Te1	0.755476 (16)	0.46663 (2)	0.414658 (7)	0.03090 (7)	
Te2	0.516642 (16)	0.42865 (2)	0.340189 (7)	0.03245 (7)	
Cl1	0.56267 (6)	0.63914 (9)	0.43435 (3)	0.03886 (19)	
Cl2	0.69532 (8)	0.57571 (11)	0.29684 (4)	0.0540 (3)	
O1	0.6465 (2)	0.1871 (3)	0.38938 (11)	0.0633 (8)	
O2	0.6185 (3)	−0.0486 (4)	0.37876 (12)	0.0844 (11)	
H2A	0.6414	−0.0550	0.3521	0.127*	
O1W	0.7110 (4)	−0.0844 (4)	0.28835 (14)	0.1222 (17)	
H1W	0.7501	−0.0326	0.2727	0.183*	
H2W	0.7111	−0.1736	0.2797	0.183*	
C11	0.8800 (2)	0.3756 (4)	0.38905 (12)	0.0360 (8)	
C12	0.8628 (3)	0.2789 (4)	0.35126 (13)	0.0462 (9)	
H12	0.7999	0.2523	0.3402	0.055*	
C13	0.9382 (3)	0.2225 (5)	0.33024 (16)	0.0623 (12)	
H13	0.9266	0.1576	0.3049	0.075*	
C14	1.0314 (3)	0.2618 (5)	0.34658 (16)	0.0659 (13)	
H14	1.0826	0.2226	0.3323	0.079*	
C15	1.0495 (3)	0.3588 (5)	0.38395 (15)	0.0577 (11)	
H15	1.1126	0.3854	0.3947	0.069*	
C16	0.9731 (3)	0.4166 (4)	0.40537 (13)	0.0449 (9)	
H16	0.9846	0.4822	0.4305	0.054*	
C21	0.7740 (2)	0.3768 (4)	0.48528 (11)	0.0338 (7)	
C22	0.6948 (3)	0.3806 (4)	0.51135 (13)	0.0456 (9)	
H22	0.6373	0.4223	0.4980	0.055*	
C23	0.7021 (3)	0.3219 (5)	0.55749 (14)	0.0557 (11)	
H23	0.6495	0.3243	0.5752	0.067*	
C24	0.7873 (3)	0.2602 (5)	0.57685 (13)	0.0549 (11)	
H24	0.7920	0.2209	0.6078	0.066*	
C25	0.8653 (3)	0.2560 (5)	0.55101 (14)	0.0528 (10)	
H25	0.9228	0.2146	0.5646	0.063*	
C26	0.8587 (3)	0.3134 (4)	0.50456 (13)	0.0439 (9)	
H26	0.9111	0.3088	0.4867	0.053*	
C31	0.8211 (2)	0.6653 (4)	0.43414 (12)	0.0343 (7)	
C32	0.8246 (3)	0.7642 (4)	0.39828 (14)	0.0495 (10)	
H32	0.8023	0.7412	0.3664	0.059*	
C33	0.8616 (3)	0.8995 (5)	0.40968 (19)	0.0657 (13)	
H33	0.8662	0.9657	0.3851	0.079*	
C34	0.8915 (3)	0.9365 (5)	0.45694 (19)	0.0623 (12)	
H34	0.9137	1.0283	0.4646	0.075*	
C35	0.8881 (3)	0.8355 (5)	0.49287 (17)	0.0613 (12)	
H35	0.9099	0.8586	0.5248	0.074*	
C36	0.8524 (3)	0.6998 (4)	0.48167 (14)	0.0483 (10)	
H36	0.8496	0.6323	0.5060	0.058*	
C41	0.5044 (2)	0.3287 (4)	0.27097 (12)	0.0343 (7)	
C42	0.5441 (3)	0.1965 (4)	0.26687 (14)	0.0520 (10)	
H42	0.5711	0.1479	0.2943	0.062*	
C43	0.5437 (3)	0.1352 (5)	0.22118 (17)	0.0609 (12)	
H43	0.5694	0.0445	0.2181	0.073*	
C44	0.5058 (3)	0.2077 (5)	0.18109 (15)	0.0591 (12)	
H44	0.5060	0.1663	0.1507	0.071*	
C45	0.4675 (3)	0.3409 (5)	0.18495 (14)	0.0568 (11)	
H45	0.4424	0.3905	0.1574	0.068*	
C46	0.4662 (3)	0.4013 (4)	0.23030 (13)	0.0432 (9)	
H46	0.4395	0.4915	0.2333	0.052*	
C51	0.4127 (2)	0.3004 (4)	0.37028 (12)	0.0376 (8)	
C52	0.4032 (3)	0.3210 (4)	0.41894 (13)	0.0483 (9)	
H52	0.4387	0.3920	0.4364	0.058*	
C53	0.3407 (3)	0.2354 (5)	0.44147 (15)	0.0601 (12)	
H53	0.3340	0.2488	0.4741	0.072*	
C54	0.2889 (3)	0.1311 (5)	0.41563 (17)	0.0664 (13)	
H54	0.2474	0.0728	0.4308	0.080*	
C55	0.2980 (4)	0.1127 (5)	0.36745 (18)	0.0728 (14)	
H55	0.2615	0.0428	0.3500	0.087*	
C56	0.3603 (3)	0.1955 (5)	0.34439 (14)	0.0556 (11)	
H56	0.3668	0.1809	0.3118	0.067*	
C61	0.4204 (3)	0.5959 (4)	0.31641 (12)	0.0358 (8)	
C62	0.3217 (3)	0.5755 (4)	0.31313 (13)	0.0457 (9)	
H62	0.2966	0.4907	0.3239	0.055*	
C63	0.2602 (3)	0.6828 (5)	0.29365 (14)	0.0563 (11)	
H63	0.1937	0.6694	0.2909	0.068*	
C64	0.2976 (4)	0.8087 (5)	0.27849 (16)	0.0660 (13)	
H64	0.2564	0.8800	0.2651	0.079*	
C65	0.3955 (4)	0.8292 (5)	0.28302 (17)	0.0683 (13)	
H65	0.4204	0.9154	0.2734	0.082*	
C66	0.4581 (3)	0.7214 (4)	0.30191 (15)	0.0541 (10)	
H66	0.5246	0.7349	0.3046	0.065*	
C91	0.6216 (3)	0.0692 (4)	0.40485 (15)	0.0510 (10)	
C92	0.5943 (5)	0.0527 (5)	0.45455 (18)	0.0861 (17)	
H92A	0.6515	0.0444	0.4771	0.129*	
H92B	0.5556	−0.0316	0.4561	0.129*	
H92C	0.5580	0.1347	0.4626	0.129*	

### Atomic displacement parameters (Å^2^)

**Table:**

	*U*^11^	*U*^22^	*U*^33^	*U*^12^	*U*^13^	*U*^23^
Te1	0.03216 (13)	0.03165 (12)	0.02833 (11)	−0.00023 (9)	0.00104 (9)	−0.00042 (9)
Te2	0.03311 (13)	0.03433 (13)	0.02905 (12)	0.00003 (9)	−0.00017 (9)	−0.00085 (9)
Cl1	0.0444 (5)	0.0338 (5)	0.0383 (4)	0.0004 (4)	0.0045 (4)	−0.0026 (3)
Cl2	0.0564 (6)	0.0542 (6)	0.0532 (6)	−0.0070 (5)	0.0135 (5)	0.0015 (5)
O1	0.085 (2)	0.0377 (16)	0.0680 (19)	−0.0048 (15)	0.0124 (16)	0.0084 (14)
O2	0.110 (3)	0.061 (2)	0.083 (3)	0.002 (2)	0.014 (2)	−0.0034 (18)
O1W	0.224 (5)	0.067 (3)	0.091 (3)	0.033 (3)	0.084 (3)	0.017 (2)
C11	0.039 (2)	0.0372 (19)	0.0330 (18)	0.0044 (15)	0.0080 (15)	0.0036 (15)
C12	0.049 (2)	0.042 (2)	0.048 (2)	0.0034 (18)	0.0061 (18)	−0.0059 (17)
C13	0.074 (3)	0.062 (3)	0.054 (3)	0.010 (2)	0.019 (2)	−0.017 (2)
C14	0.067 (3)	0.072 (3)	0.063 (3)	0.027 (3)	0.027 (2)	0.007 (2)
C15	0.041 (2)	0.074 (3)	0.059 (3)	0.011 (2)	0.012 (2)	0.011 (2)
C16	0.043 (2)	0.051 (2)	0.041 (2)	0.0033 (18)	0.0070 (17)	0.0045 (17)
C21	0.042 (2)	0.0333 (18)	0.0258 (16)	−0.0018 (15)	0.0037 (14)	−0.0012 (14)
C22	0.046 (2)	0.053 (2)	0.039 (2)	0.0079 (18)	0.0082 (17)	0.0061 (17)
C23	0.057 (3)	0.071 (3)	0.043 (2)	0.010 (2)	0.0200 (19)	0.012 (2)
C24	0.074 (3)	0.058 (3)	0.033 (2)	0.009 (2)	0.006 (2)	0.0118 (18)
C25	0.051 (2)	0.063 (3)	0.042 (2)	0.013 (2)	−0.0044 (19)	0.0086 (19)
C26	0.039 (2)	0.051 (2)	0.043 (2)	0.0064 (17)	0.0094 (16)	0.0040 (17)
C31	0.0308 (18)	0.0336 (19)	0.0383 (19)	0.0003 (14)	0.0037 (14)	−0.0060 (15)
C32	0.055 (2)	0.039 (2)	0.052 (2)	−0.0055 (19)	−0.0016 (19)	0.0029 (18)
C33	0.065 (3)	0.043 (3)	0.087 (4)	−0.011 (2)	0.004 (3)	0.012 (2)
C34	0.053 (3)	0.039 (2)	0.096 (4)	−0.012 (2)	0.011 (3)	−0.018 (2)
C35	0.056 (3)	0.065 (3)	0.065 (3)	−0.017 (2)	0.009 (2)	−0.029 (2)
C36	0.050 (2)	0.052 (2)	0.044 (2)	−0.0111 (19)	0.0073 (18)	−0.0054 (18)
C41	0.0322 (18)	0.0373 (19)	0.0339 (18)	−0.0061 (15)	0.0059 (14)	−0.0033 (14)
C42	0.062 (3)	0.048 (2)	0.045 (2)	0.010 (2)	0.0047 (19)	−0.0006 (18)
C43	0.066 (3)	0.047 (3)	0.072 (3)	0.005 (2)	0.019 (2)	−0.022 (2)
C44	0.070 (3)	0.067 (3)	0.043 (2)	−0.009 (2)	0.017 (2)	−0.021 (2)
C45	0.074 (3)	0.060 (3)	0.035 (2)	−0.010 (2)	−0.0005 (19)	−0.0055 (19)
C46	0.050 (2)	0.041 (2)	0.037 (2)	−0.0042 (17)	−0.0004 (17)	−0.0051 (16)
C51	0.039 (2)	0.038 (2)	0.0369 (19)	0.0005 (15)	0.0055 (15)	0.0045 (15)
C52	0.058 (3)	0.049 (2)	0.040 (2)	−0.0018 (19)	0.0103 (18)	−0.0023 (17)
C53	0.074 (3)	0.064 (3)	0.046 (2)	−0.008 (2)	0.024 (2)	0.003 (2)
C54	0.074 (3)	0.060 (3)	0.072 (3)	−0.016 (2)	0.034 (3)	0.000 (2)
C55	0.077 (3)	0.071 (3)	0.075 (3)	−0.037 (3)	0.027 (3)	−0.022 (3)
C56	0.060 (3)	0.064 (3)	0.044 (2)	−0.019 (2)	0.0124 (19)	−0.012 (2)
C61	0.040 (2)	0.037 (2)	0.0300 (17)	0.0049 (15)	0.0004 (15)	−0.0021 (14)
C62	0.044 (2)	0.049 (2)	0.043 (2)	0.0060 (18)	0.0008 (17)	0.0014 (17)
C63	0.048 (3)	0.066 (3)	0.053 (2)	0.014 (2)	−0.0031 (19)	−0.003 (2)
C64	0.072 (3)	0.060 (3)	0.063 (3)	0.027 (3)	−0.007 (2)	−0.001 (2)
C65	0.080 (4)	0.040 (3)	0.085 (3)	0.007 (2)	0.009 (3)	0.006 (2)
C66	0.053 (3)	0.042 (2)	0.068 (3)	0.0009 (19)	0.007 (2)	0.001 (2)
C91	0.060 (3)	0.036 (2)	0.056 (3)	0.0077 (19)	0.001 (2)	0.0035 (18)
C92	0.141 (5)	0.057 (3)	0.065 (3)	0.008 (3)	0.035 (3)	0.009 (2)

### Geometric parameters (Å, º)

**Table:**

Te1—C11	2.129 (3)	C33—H33	0.9300
Te1—C21	2.124 (3)	C34—C35	1.380 (6)
Te1—C31	2.116 (3)	C34—H34	0.9300
Te1—Cl1	3.2366 (9)	C35—C36	1.387 (6)
Te1—Cl2	3.4407 (11)	C35—H35	0.9300
Te1—O1	3.067 (3)	C36—H36	0.9300
Te2—C41	2.129 (4)	C41—C42	1.366 (5)
Te2—C51	2.126 (4)	C41—C46	1.373 (5)
Te2—C61	2.118 (4)	C42—C43	1.393 (5)
Te2—Cl1	3.2802 (9)	C42—H42	0.9300
Te2—Cl2	3.2007 (11)	C43—C44	1.359 (6)
Te2—O1	3.113 (3)	C43—H43	0.9300
O1—C91	1.249 (5)	C44—C45	1.366 (6)
O2—C91	1.318 (5)	C44—H44	0.9300
O2—H2A	0.8430	C45—C46	1.384 (5)
O1W—H1W	0.8801	C45—H45	0.9300
O1W—H2W	0.8691	C46—H46	0.9300
C11—C16	1.380 (5)	C51—C56	1.377 (5)
C11—C12	1.387 (5)	C51—C52	1.388 (5)
C12—C13	1.367 (5)	C52—C53	1.385 (5)
C12—H12	0.9300	C52—H52	0.9300
C13—C14	1.376 (6)	C53—C54	1.369 (6)
C13—H13	0.9300	C53—H53	0.9300
C14—C15	1.381 (6)	C54—C55	1.371 (6)
C14—H14	0.9300	C54—H54	0.9300
C15—C16	1.388 (5)	C55—C56	1.376 (6)
C15—H15	0.9300	C55—H55	0.9300
C16—H16	0.9300	C56—H56	0.9300
C21—C26	1.375 (5)	C61—C66	1.367 (5)
C21—C22	1.389 (5)	C61—C62	1.383 (5)
C22—C23	1.388 (5)	C62—C63	1.390 (5)
C22—H22	0.9300	C62—H62	0.9300
C23—C24	1.374 (5)	C63—C64	1.375 (6)
C23—H23	0.9300	C63—H63	0.9300
C24—C25	1.372 (6)	C64—C65	1.370 (6)
C24—H24	0.9300	C64—H64	0.9300
C25—C26	1.392 (5)	C65—C66	1.397 (6)
C25—H25	0.9300	C65—H65	0.9300
C26—H26	0.9300	C66—H66	0.9300
C31—C32	1.365 (5)	C91—C92	1.483 (6)
C31—C36	1.381 (5)	C92—H92A	0.9600
C32—C33	1.391 (6)	C92—H92B	0.9600
C32—H32	0.9300	C92—H92C	0.9600
C33—C34	1.376 (6)		
			
C31—Te1—C21	96.21 (13)	C33—C32—H32	120.2
C31—Te1—C11	95.27 (13)	C34—C33—C32	120.7 (4)
C21—Te1—C11	97.65 (13)	C34—C33—H33	119.7
Cl1—Te1—Cl2	84.04 (2)	C32—C33—H33	119.7
Cl1—Te1—O1	93.72 (12)	C33—C34—C35	119.1 (4)
Cl2—Te1—O1	88.56 (12)	C33—C34—H34	120.4
Cl1—Te1—C11	169.01 (13)	C35—C34—H34	120.4
Cl1—Te1—C21	93.25 (13)	C34—C35—C36	120.4 (4)
Cl1—Te1—C31	82.03 (13)	C34—C35—H35	119.8
Cl2—Te1—C11	85.40 (13)	C36—C35—H35	119.8
Cl2—Te1—C21	170.99 (13)	C35—C36—C31	119.6 (4)
Cl2—Te1—C31	91.93 (13)	C35—C36—H36	120.2
O1—Te1—C11	89.08 (13)	C31—C36—H36	120.2
O1—Te1—C21	83.03 (13)	C42—C41—C46	120.2 (3)
O1—Te1—C31	175.64 (13)	C42—C41—Te2	118.8 (3)
C61—Te2—C51	95.97 (14)	C46—C41—Te2	120.6 (3)
C61—Te2—C41	93.47 (13)	C41—C42—C43	119.3 (4)
C51—Te2—C41	96.86 (13)	C41—C42—H42	120.3
Cl1—Te2—Cl2	87.27 (2)	C43—C42—H42	120.3
Cl1—Te2—O1	92.02 (12)	C44—C43—C42	120.2 (4)
Cl2—Te2—O1	92.23 (12)	C44—C43—H43	119.9
Cl1—Te2—C41	166.75 (13)	C42—C43—H43	119.9
Cl1—Te2—C51	96.04 (13)	C43—C44—C45	120.7 (4)
Cl1—Te2—C61	82.17 (13)	C43—C44—H44	119.7
Cl2—Te2—C41	80.47 (13)	C45—C44—H44	119.7
Cl2—Te2—C51	170.50 (13)	C44—C45—C46	119.4 (4)
Cl2—Te2—C61	93.29 (13)	C44—C45—H45	120.3
O1—Te2—C41	93.44 (13)	C46—C45—H45	120.3
O1—Te2—C51	78.78 (13)	C41—C46—C45	120.2 (4)
O1—Te2—C61	171.78 (13)	C41—C46—H46	119.9
Te1—Cl1—Te2	69.86 (2)	C45—C46—H46	119.9
Te1—Cl2—Te2	68.26 (2)	C56—C51—C52	120.3 (3)
Te1—O1—Te2	74.28 (12)	C56—C51—Te2	122.9 (3)
C91—O2—H2A	123.5	C52—C51—Te2	116.8 (3)
H1W—O1W—H2W	111.9	C53—C52—C51	119.7 (4)
C16—C11—C12	120.3 (3)	C53—C52—H52	120.1
C16—C11—Te1	123.5 (3)	C51—C52—H52	120.1
C12—C11—Te1	115.9 (3)	C54—C53—C52	119.9 (4)
C13—C12—C11	120.1 (4)	C54—C53—H53	120.1
C13—C12—H12	120.0	C52—C53—H53	120.1
C11—C12—H12	120.0	C55—C54—C53	120.0 (4)
C12—C13—C14	120.0 (4)	C55—C54—H54	120.0
C12—C13—H13	120.0	C53—C54—H54	120.0
C14—C13—H13	120.0	C54—C55—C56	121.2 (4)
C13—C14—C15	120.5 (4)	C54—C55—H55	119.4
C13—C14—H14	119.7	C56—C55—H55	119.4
C15—C14—H14	119.7	C51—C56—C55	119.0 (4)
C14—C15—C16	119.7 (4)	C51—C56—H56	120.5
C14—C15—H15	120.2	C55—C56—H56	120.5
C16—C15—H15	120.2	C66—C61—C62	120.9 (4)
C11—C16—C15	119.4 (4)	C66—C61—Te2	118.4 (3)
C11—C16—H16	120.3	C62—C61—Te2	120.6 (3)
C15—C16—H16	120.3	C61—C62—C63	119.4 (4)
C26—C21—C22	120.4 (3)	C61—C62—H62	120.3
C26—C21—Te1	122.6 (2)	C63—C62—H62	120.3
C22—C21—Te1	116.9 (3)	C64—C63—C62	120.0 (4)
C21—C22—C23	119.6 (4)	C64—C63—H63	120.0
C21—C22—H22	120.2	C62—C63—H63	120.0
C23—C22—H22	120.2	C63—C64—C65	120.1 (4)
C24—C23—C22	119.8 (4)	C63—C64—H64	119.9
C24—C23—H23	120.1	C65—C64—H64	119.9
C22—C23—H23	120.1	C64—C65—C66	120.4 (4)
C23—C24—C25	120.6 (4)	C64—C65—H65	119.8
C23—C24—H24	119.7	C66—C65—H65	119.8
C25—C24—H24	119.7	C61—C66—C65	119.1 (4)
C24—C25—C26	120.2 (4)	C61—C66—H66	120.4
C24—C25—H25	119.9	C65—C66—H66	120.4
C26—C25—H25	119.9	O1—C91—O2	122.9 (4)
C21—C26—C25	119.4 (3)	O1—C91—C92	121.6 (4)
C21—C26—H26	120.3	O2—C91—C92	115.5 (4)
C25—C26—H26	120.3	C91—C92—H92A	109.5
C32—C31—C36	120.4 (3)	C91—C92—H92B	109.5
C32—C31—Te1	117.4 (3)	H92A—C92—H92B	109.5
C36—C31—Te1	122.0 (3)	C91—C92—H92C	109.5
C31—C32—C33	119.6 (4)	H92A—C92—H92C	109.5
C31—C32—H32	120.2	H92B—C92—H92C	109.5

### Hydrogen-bond geometry (Å, º)

**Table:**

*D*—H···*A*	*D*—H	H···*A*	*D*···*A*	*D*—H···*A*
O2—H2*A*···O1*W*	0.84	2.13	2.972 (5)	174
O1*W*—H1*W*···Cl2^i^	0.88	2.38	3.205 (4)	155
O1*W*—H2*W*···Cl2^ii^	0.87	2.41	3.200 (4)	152

Symmetry codes: (i) −*x*+3/2, *y*−1/2, −*z*+1/2; (ii) *x*, *y*−1, *z*.
